# Sudden Death Caused by Gastroesophageal Varices Rupture: Insights From an Autopsy-Based Case Series Unraveling the Pathological Events

**DOI:** 10.7759/cureus.46166

**Published:** 2023-09-28

**Authors:** Arushi Verma, Raviprakash Meshram, Ravi H Phulware, Shailesh Parate, Vikas Vaibhav

**Affiliations:** 1 Forensic Medicine and Toxicology, All India Institute of Medical Sciences, Rishikesh, IND; 2 Pathology and Laboratory Medicine, All India Institute of Medical Sciences, Rishikesh, IND

**Keywords:** forensic pathology, sudden death, portal hypertension, gastroesophageal varices, gastroesophageal bleeding

## Abstract

Sudden death is characterized by natural yet unexpected death, typically occurring within 24 hours from the onset of the patient's symptoms. While the majority of sudden deaths stem from cardiac issues/causes, there are instances where non-cardiac factors are at play. One such scenario involves hemorrhage from ruptured esophageal varices, a complication that stems from portal hypertension. Portal hypertension can manifest due to a range of pre-hepatic, hepatic, and post-hepatic conditions, with liver cirrhosis being the primary culprit. Although sudden death cases linked to the gastrointestinal system are relatively rare, the rupture of gastroesophageal varices, precipitating severe morbidity and a high mortality rate, represents a life-threatening condition. In this context, we present a case series encompassing five instances of sudden natural deaths arising from the rupture of gastroesophageal varices.

## Introduction

Forensic pathologists regularly encounter cases of abrupt natural deaths. The World Health Organization (WHO) defines sudden death as "death occurring within 24 hours from the onset of acute signs and symptoms," but it is worth noting that many clinicians and pathologists narrow this window to one hour from the onset of symptoms [1-4]. In adults and the elderly, the predominant causes of sudden natural deaths are cardiac in nature, with coronary artery disease leading to coronary thrombosis or myocardial infarction being the most common culprits [5-7]. Among the non-cardiac factors, the central nervous and respiratory systems are the primary contributors, followed by the gastrointestinal (GI) and peripheral vascular systems [8]. Although less frequent, acute hemorrhage in the upper GI tract is a notable cause of sudden natural death. Ruptured esophageal varices are the major cause of upper GI hemorrhage in cirrhotic patients, accounting for approximately 70% of cases [9]. These varices develop as complications of portal hypertension, stemming from various factors, namely, pre-hepatic, hepatic, and post-hepatic in origin. The leading cause of portal hypertension is decompensated liver cirrhosis. The mortality rate associated with ruptured esophageal varices ranges from 5% to 50% in patients with liver cirrhosis [10]. In this report, we present five cases of sudden natural death attributed to ruptured esophageal varices, as determined during medico-legal autopsy.

## Case presentation

Five cases were received over a span of three years for medico-legal autopsy at the mortuary, Department of Forensic Medicine and Toxicology, AIIMS Rishikesh. One case in December 2021, three cases in January 2023, and one case in February 2023. All the individuals were male with ages ranging between 30 and 70 years. Among these cases, two were brought dead to the hospital, while the remaining three succumbed during their hospitalization within seven to 12 hours of admission. Out of these three inpatients, two had a history of hematemesis, and one had a history of chest pain. Chronic alcohol consumption was documented in only two cases and no history of alcohol intake was available in the rest of the cases.

During the autopsy, external examination revealed that three of the individuals were of average build, while the other two were thin build. Notably, one of the cases found unconscious in a bathroom and declared brought dead at the hospital presented with multiple fresh injuries, indicating antemortem trauma.

**Figure 1:**
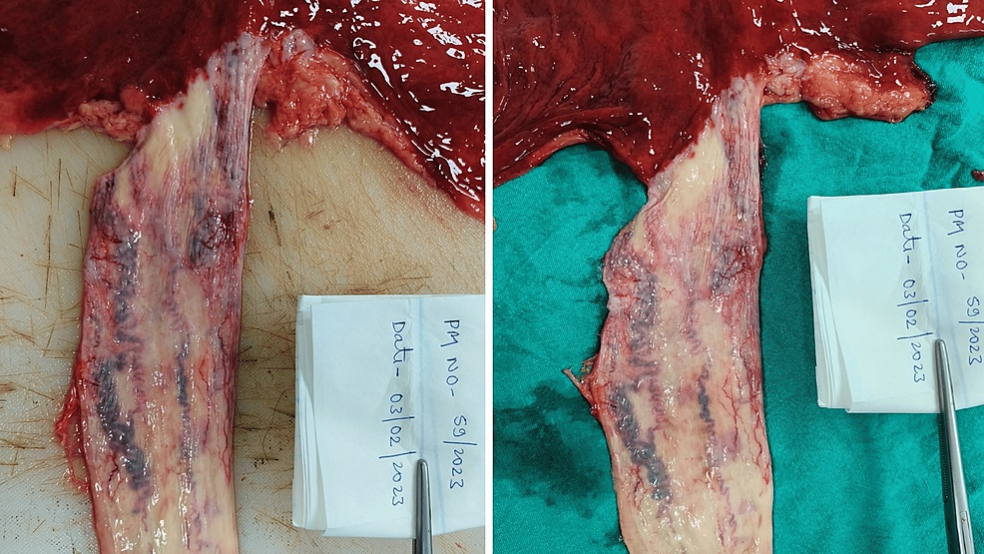
Esophagus gross showing dilated and tortuous vessels at the lower 1/3rd part surrounded by patches of erosion and blood staining (Case 5).

**Figure 2:**
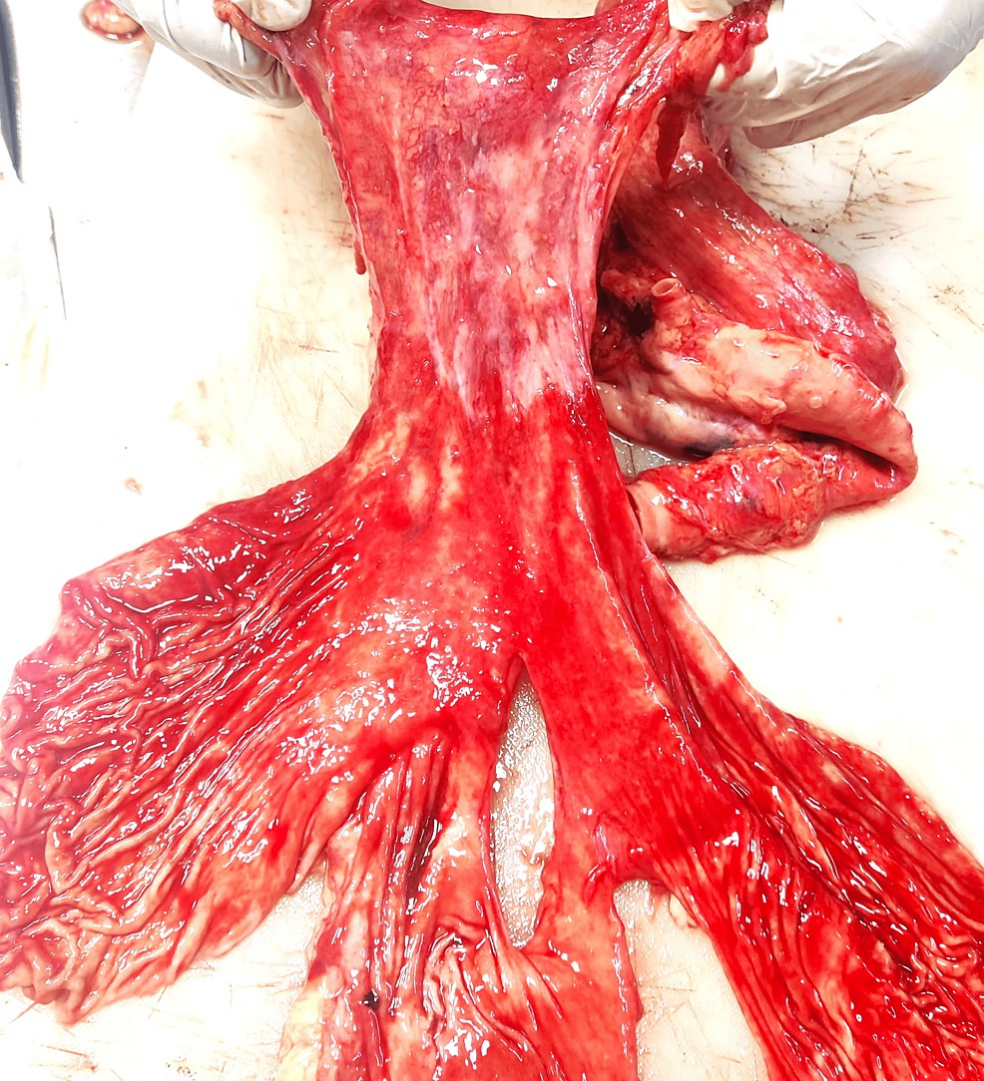
Multiple, bluish, dilated, and tortuous submucosal blood vessels at the lower 1/3rd of the esophagus with congested mucosa (Case 4).

**Figure 3:**
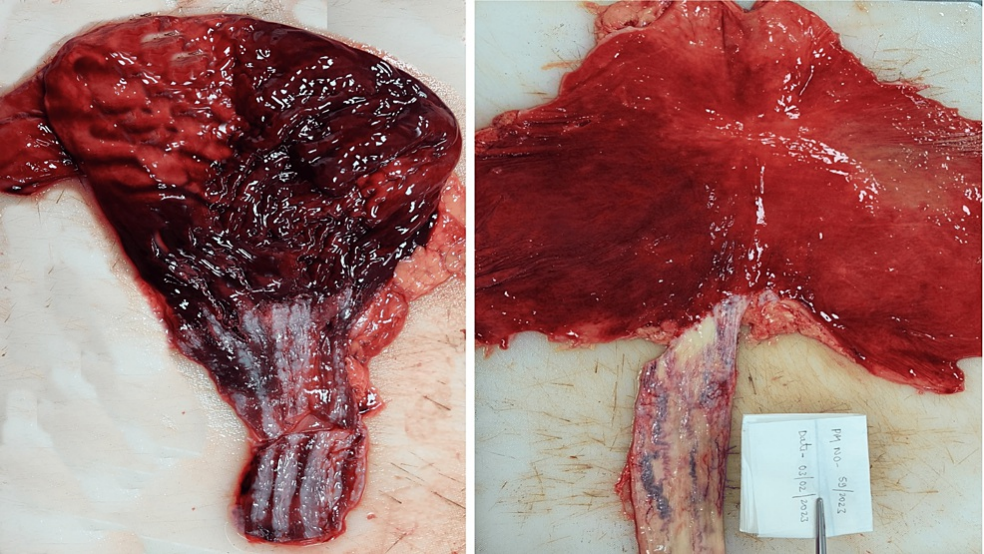
Gastroesophageal mucosa. Alternate bands of colored and pale areas (watermelon appearance) and multiple tortuous vessels present over the cardiac end of the stomach with blood-stained mucosa (Cases 1 & 5).

Upon internal examination, significant morphological changes with pathological implications were identified in various organ systems, primarily in the hepatobiliary, gastrointestinal, lymphoreticular, and respiratory systems. In the gastrointestinal system, dilated and tortuous submucosal vessels, as well as congested mucosa, were observed in the lower third of the esophagus in four cases. In one case, dilated and tortuous submucosal vessels were found at the gastroesophageal junction and near the cardiac end of the stomach, accompanied by congested esophageal mucosa (Figures 1-3, cases 4 and 5). All five cases showed the presence of altered blood in the stomach ranging from 10 ml to 2000 ml.

**Figure 4:**
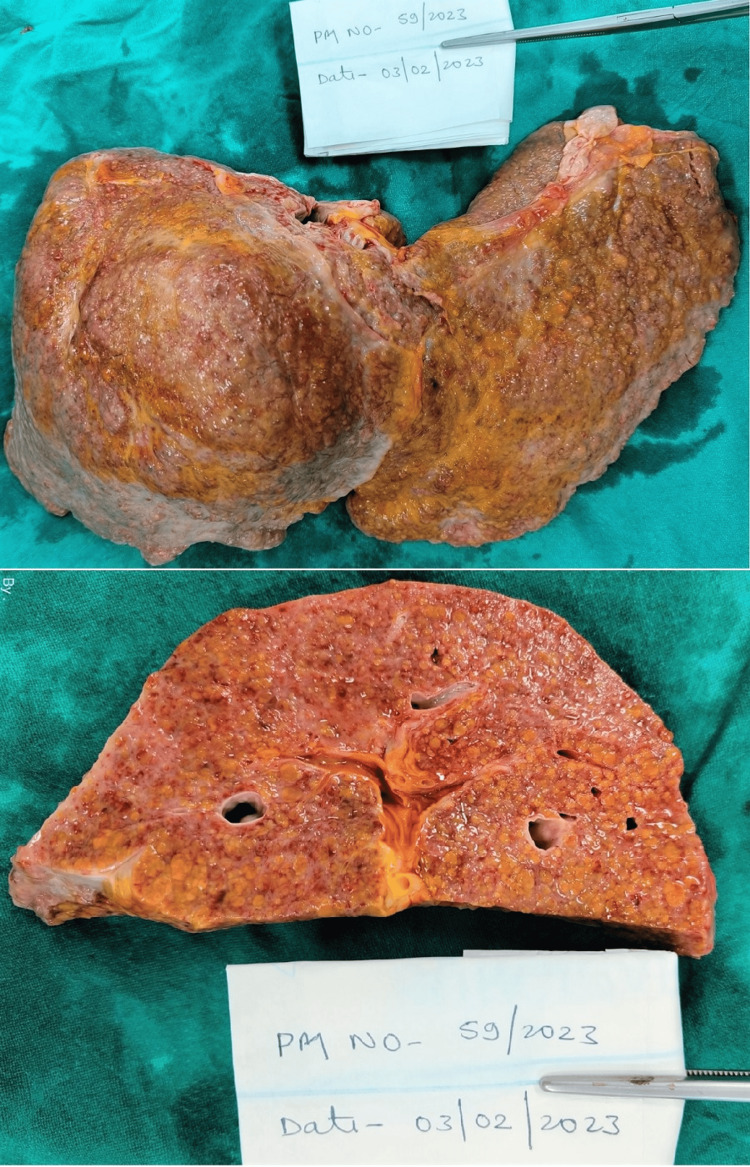
Liver gross showing macronodular lesions along with yellowish discoloration (Case 5).

Hepatomegaly was observed in three cases while liver weight was normal in two cases (1477 +/- 370.52 gm) [11]. Two of these cases showed firm liver consistency with yellowish discoloration on both the outer and cut surfaces, along with the presence of micro and macro nodules across the entire external surface. In one case, fatty changes in the form of yellowish discolored areas were detected on the liver's outer and cut surfaces (Figure 4, case 5).

**Figure 5:**
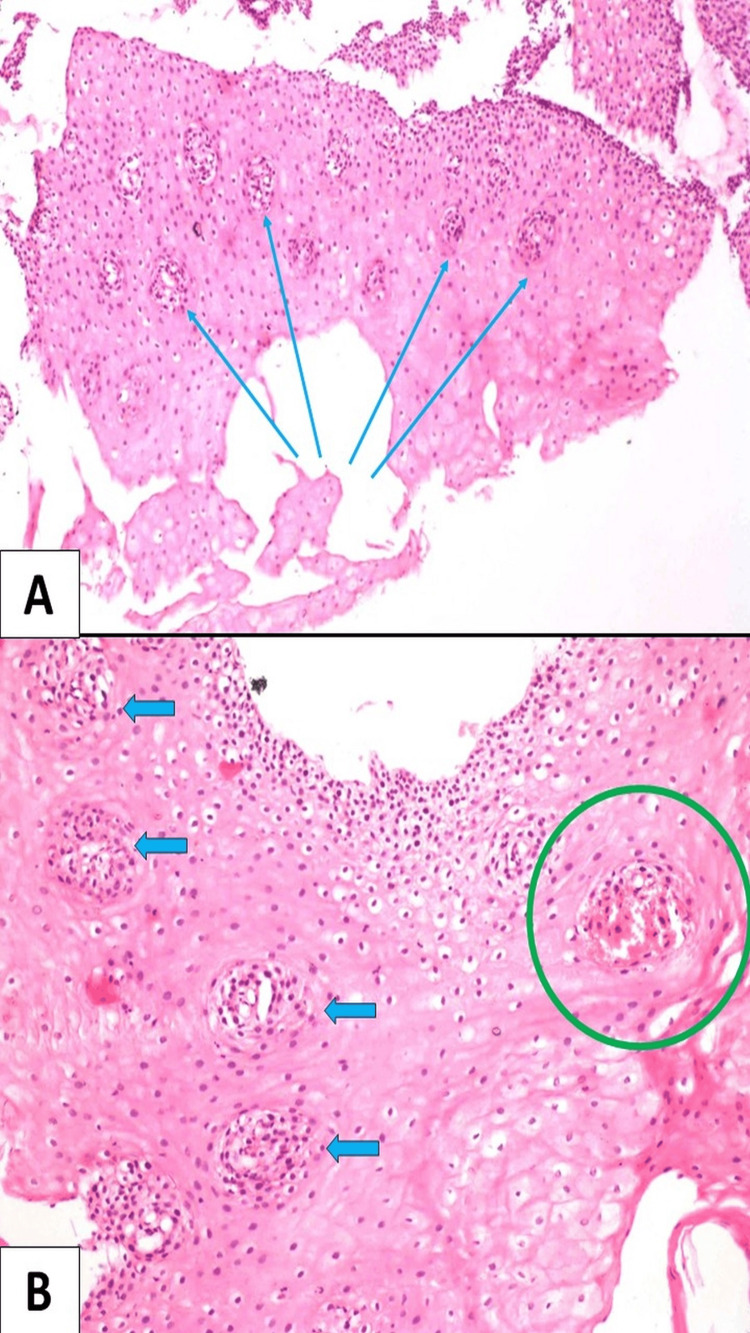
(A) Hematoxylin and eosin (H&E)-stained sections (x100) show hyperplastic stratified squamous epithelium of esophageal mucosa displaying basal cell hyperplasia (blue arrow) and scattered inflammatory cells. (B) Higher magnification (H&E, x400) shows congested blood vessels and extravasation of red blood cells (green circle) (Case 5).

**Figure 6:**
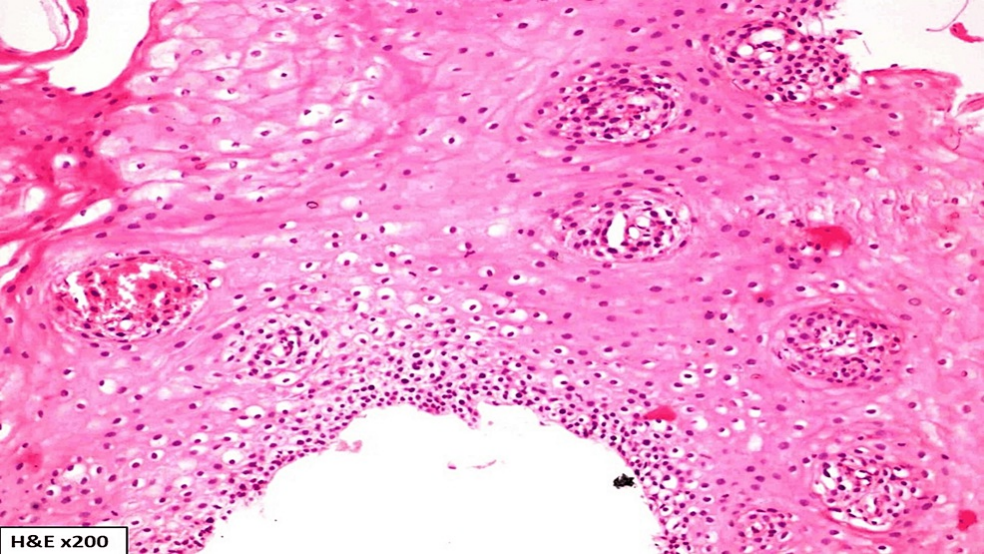
Hematoxylin and eosin (H&E)-stained sections (x200) show esophageal mucosa displaying features of gastroesophageal reflux disease in the form of basal cell hyperplasia, elongated rete ridges, and blood lake formation with interspersed inflammatory cells (Case 4).

Splenomegaly was present in two cases; spleen weight was within the normal range in one case (154 +/- 74.63 gm) and reduced in the remaining two cases [11]. Lung weight was increased, and both lungs were edematous in three cases, in the remaining two cases, it was within a normal range (right lung = 499.4 +/- 207.5 gm; left lung = 407.5gm +/- 128.66 gm) [11]. Brain injury attributable to blunt force or surface impact was present in one case. Atheromatous changes resulting in significant narrowing of the left main coronary artery were observed in one case. The cause of death was hemorrhagic shock resulting from the rupture of esophageal varices in most of the cases; however, there were other contributory factors such as brain injury, coronary artery disease, and chronic liver disease. Following the postmortem examination, tissue samples were retained and forwarded for histopathological examination. Upon histopathological analysis of the esophagus, the following observations were made: under examination with hematoxylin and eosin (H&E) staining, the sections revealed hyperplastic stratified squamous epithelium in the esophageal mucosa. This was characterized by basal cell hyperplasia and the presence of dispersed inflammatory cells when viewed at a 100x magnification (Figure 5, case 5). When viewed at higher magnification (H&E, x400), the examination disclosed congested blood vessels accompanied by the leakage of red blood cells (RBCs) (Figure 5, case 5). Further, on histopathological examination, H&E-stained sections (x200) show esophageal mucosa displaying features of gastroesophageal reflux disease (GERD) in the form of basal cell hyperplasia, elongated rete ridges, and blood lake formation with interspersed inflammatory cells (Figure 6, case 4).

Table 1 provides an overview of the demographic profile and clinical features of all five cases, while Table 2 summarizes their autopsy findings.

**Table 1:** Demographic profile and clinical features of all five cases. NR = not recordable; NA = not available; WNL = within normal limits.

Parameter	Case 1	Case 2	Case 3	Case 4	Case 5
Age	31 years	40 years	About 70 years	62 years	56 years
Sex	Male	Male	Male	Male	Male
History	Chest pain	Found unconscious inside the bathroom, brought dead to AIIMS Rishikesh	Brought dead to SPS Hospital, Rishikesh	Bloody vomiting	Bloody vomiting
Time duration between onset of symptoms and death	7-8 hours	1-2 hours	Not available	11-12 hours	About 12 hours
History of alcohol intake	Occasional drinker	Heavy drinker for the past 4-5 years	Not available	Not available	Not available
Vitals					
Heart rate	40 beats/min	NR	NR	100 beats/min	112 beats/min
Oxygen saturation (SpO_2_)	96% (Ambu bag)	NR	NR	88% (room air)	100% (room air)
Blood pressure	70/40mm Hg (on inotropes)	NR	NR	80/64 mm Hg	62/40 mm Hg
Glasgow Coma Scale	Critical	NR	NR	E1V1M1	E4V5M6
Investigations					
ECG	WNL	NA	NA	Not available	Not available
ECHO	WNL	NA	NA	Not available	Not available
Others	Not available	NA	NA	Not available	Not available

**Table 2:** Autopsy findings of all five cases. NAD = no abnormality detected; SDH = subdural hemorrhage; SAH = subarachnoid hemorrhage; LADA = left anterior descending artery; LMCA = left main coronary artery; RCA = right coronary artery; LCX = left circumflex artery.

Autopsy findings	Case 1	Case 2	Case 3	Case 4	Case 5
External findings	Average built, dried blood present at nostrils	Average built, multiple fresh antemortem injuries over body	Thin built with sunken abdomen	Average built, yellowish discoloration of sclera present. Blood oozing out from both nostrils, mouth, and anus	Thin built with sunken abdomen
Esophagus	Congested mucosa	Dilated, tortuous, eroded vessels at the lower end with brownish-black staining of the mucosal lining	Multiple, bluish, dilated, and tortuous submucosal blood vessels at the lower 1/3^rd^ of the esophagus with congested mucosa	Multiple, bluish, dilated, and tortuous submucosal blood vessels at the lower 1/3^rd^ with congested and eroded mucosa (Figure 2)	Multiple, dilated, and tortuous vessels over the lower 1/3^rd^ of the esophagus, surrounded by patches of erosion and blood staining at places (Figure 1)
Peritoneal cavity	Contains 1000 ml of blood	NAD	NAD	NAD	NAD
Stomach	Content - 950 ml of reddish-brown colored blood mixed with semi-digested food material without any abnormal smell. Mucosa - congested with multiple hemorrhagic patches at places. Multiple, prominent, dilated tortuous veins at the gastroesophageal junction and near the cardiac end (Figure 3).	Content - 1000 ml of blood without any abnormal smell. Mucosa - dilated, tortuous, eroded vessels at the cardiac end of the stomach with brownish-black staining of the mucosal lining	Content - 650 ml of black-colored altered blood with blood clots. Mucosa - congested with prominent rugae and blood-mixed mucoid material sticking to the wall	Content - 10 ml of blood mixed with mucoid material. Mucosa - congested with ecchymotic patches at places	About 2000 ml of blood without any abnormal smell. Mucosa - multiple bands of colored and pale (watermelon appearance) (Figure 3), multiple tortuous vessels present over the cardiac end of the stomach with blood-stained mucosa
Liver	Weight - 1540 gm, pale and friable	Weight - 1780 gm. Enlarged and pale, fatty changes present	Weight - 890 gm. Pale	Weight - 1130 gm. Yellowish in color and firm consistency. Multiple irregular nodules varying in size from 0.5 cm x 0.5 cm to 1 cm x 0.5 cm were scattered all over the surface, separated by fibrotic tissue. On the cut section, yellowish, irregular parenchyma was present throughout (cirrhosis)	1870 gm. Enlarged and pale. Multiple macro-nodules ranging in size from 0.9 cm x 0.8 cm to 0.3 cm x 0.2 cm were present all over with yellowish discoloration. On the cut section, firm consistency with a nodular surface (cirrhosis) (Figure 4)
Spleen	Weight - 240gm, pale and enlarged	Weight - 60 gm. Reduced in size and pale	Weight - 20 gm. Reduced in size and pale	Weight - 120 gm, NAD	Weight - 320 gm. Enlarged and pale
Other relevant findings	Both lungs were edematous. All organs were pale	Brain - 1400 gm, edematous and congested. SDH + SAH - right cerebral hemisphere & left frontal lobe. All organs pale	Both lungs were edematous. Cortico-medullary junction was indistinct in both kidneys. All organs were pale	Heart - weight 300 gm, congested. Whitish scar tissue - posterior surface. Atheromatous changes and calcification - all coronaries. LMCA - >90% narrowing, 0.5 cm distal to origin; LADA - about 40-50% narrowing throughout with multiple collateral vessel formation; RCA & LCX- about 10-20% narrowing throughout	Both lungs were edematous; the cortico-medullary junction is indistinct in both kidneys. All organs were pale
Cause of death	Hemorrhagic shock d/t rupture of gastroesophageal varices, which is a natural cause	The combined effect of hemorrhagic shock and brain injury consequent upon variceal bleeding and blunt force/surface impact to the head, respectively	Hemorrhagic shock d/t rupture of esophageal varices, which is a natural cause	Coronary artery disease in a pre-existing case of chronic liver disease, which is a natural cause	Hemorrhagic shock consequent upon lower esophageal variceal bleeding, which is a natural cause

## Discussion

The portal vein is formed by the union of the superior mesenteric vein, responsible for draining blood from the splanchnic circulation, and the splenic vein [12]. Portal hypertension occurs when there is an increase in vascular resistance, leading to a defined increase in the hepatic venous pressure gradient (HVPG) beyond 5 mmHg [10,12]. This increase in vascular resistance can occur at various levels: prehepatic, such as in cases of portal and splenic vein thrombosis; intrahepatic, as seen in liver cirrhosis; and posthepatic, including conditions like Budd-Chiari syndrome, with cirrhosis being the major cause of portal hypertension [12].

Due to the elevated pressure within the portal vein, portosystemic collaterals develop as a compensatory mechanism to mitigate the increased resistance. Among these portosystemic collaterals, the most significant are the esophageal varices, which pose a substantial risk of upper GI bleeding and subsequent mortality. Esophageal varices are dilated submucosal veins in the distal esophagus that connect the portal and systemic circulations [13]. The risk of developing esophageal varices and their subsequent rupture significantly increases when the HVPG exceeds 10 mmHg [12]. Annually, approximately 5-15% of individuals with liver cirrhosis develop esophageal varices. Alcoholic liver disease is the most prominent among the many causes of liver cirrhosis [10]. Esophageal varices present a significant risk of hemorrhage and are responsible for 70% of cases of upper GI hemorrhage in these patients [9]. The mortality rate associated with ruptured esophageal varices ranges from 5% to 50% in patients with liver cirrhosis [10,14]. In our case series, liver cirrhosis was detected in only two cases, one case exhibited fatty changes without cirrhosis, and in two cases, the liver appeared entirely normal. This underscores the fact that while liver cirrhosis is the most common cause of portal hypertension, it can also result from various other prehepatic, hepatic, and posthepatic conditions such as portal vein thrombosis, sarcoidosis, and Budd-Chiari syndrome, among others.

It is important to emphasize that the development and rupture of varices necessitate an elevation in portal pressure. However, local factors, such as variceal wall tension, also play a significant role in predicting the risk of variceal hemorrhage. The mechanism behind the rupture of esophageal varices can be understood through Laplace's law [14]. Rupture occurs when the wall tension of esophageal varices exceeds their elastic limit, leading to hemorrhage. Wall tension is directly related to intravariceal pressure (which is associated with portal pressure) and variceal diameter, and inversely related to variceal wall thickness. Consequently, large varices with thin walls pose a greater risk of bleeding compared to smaller varices with thicker walls. Other significant risk factors for variceal rupture include decompensated cirrhosis, the presence of red color markings on the varices during endoscopy, and active alcohol consumption. Variceal rupture frequently occurs at the gastroesophageal junction, where varices are relatively superficial and have thin walls [13,14]. The chances of rupture are 5% for small varices and 15% for large varices within one year of developing gastroesophageal varices [10]. In our case series, variceal rupture was observed in the lower one-third of the esophagus in four cases, whereas in one case, it occurred at the gastroesophageal junction and the cardiac region of the stomach.

Clinically, varices are often first indicated by episodes of GI bleeding, which can manifest as hematemesis, hematochezia, and/or melena. Occult bleeding (anemia) is infrequent. Patients may present with signs of active bleeding, such as hypotension and tachycardia. Additionally, these patients may have a history of alcoholism, weight loss, anorexia, jaundice, pruritus, and altered mental status [13]. Upon physical examination, findings may include splenomegaly, an enlarged or cirrhotic (small and firm) liver, ascites, visible periumbilical collateral circulation (caput medusae), and peripheral stigmata of alcohol abuse, such as spider angiomas on the chest/back, palmar erythema, testicular atrophy, gynecomastia, and palmar erythema [13]. Typically, individuals with this condition are found dead with substantial amounts of vomited blood nearby and often in unusual body positions, leading to suspicions of foul play. Consequently, a postmortem examination becomes necessary in all such cases [15]. In our current case series, a history of hematemesis was documented in only two of the cases, while two cases were brought dead, and one case presented a history of chest pain.

A study conducted by Tsokos and Türk over five years, focusing on 45 cases of sudden death due to variceal bleeding, unveiled an overall incidence of 0.75%. This occurrence exhibited a male preponderance, with a mean age of approximately 50 years. All deaths occurred outside of hospital settings and 93% of cases had a positive history of alcohol consumption [16]. These findings are consistent with our current case series, where all five cases were male, and the mean age was around 52 years. Among the five cases, only two were brought dead to the hospital, and for the three cases that died in the hospital, the time elapsed between symptom onset and death ranged from seven to 12 hours.

It is noteworthy that the risk of variceal bleeding can be significantly mitigated through upper GI endoscopy, considered the gold standard for screening esophageal varices. Endoscopy allows for the assessment of variceal size and the identification of varices with thin walls that are at high risk of bleeding, such as those exhibiting red wheals, red spots, or diffuse redness [14,15].

## Conclusions

The primary cause of death in the majority of our cases was identified as hemorrhagic shock resulting from the rupture of gastroesophageal varices. This highlights the significance of hemorrhagic shock due to gastroesophageal variceal rupture as a significant contributor to sudden deaths. All cases of sudden death necessitate a thorough investigation, including the conduct of a medico-legal autopsy, to determine the exact cause of death. Unfortunately, in many developing countries, numerous cases of sudden natural death do not undergo comprehensive postmortem examinations, which increases the risk of erroneously attributing the death to cardiac causes. Postmortem examination in these cases can provide invaluable insights into the cause of death, contributing to a better understanding of and prevention of such occurrences. It is advisable to mandate screening for liver conditions or cirrhotic changes in individuals with a history of chronic alcohol consumption. Additionally, prophylactic measures should be implemented in patients with liver cirrhosis to mitigate the risk of sudden death due to rupture of gastroesophageal varices.
